# Hospital Progress and Finance

**Published:** 1924-02

**Authors:** 


### HOSPITAL PROGRESS AND FINANCE.

### HOSPITAL SAVING ASSOCIATION.

PRESIDING over the National Federation of Employees' Approved Societies, Mr. H. Lesser stated that several societies had adopted the Hospital Saving Association's scheme. The workmen contributed in groups, each of which had its own secretary, who provided the members with a certificate when he or his dependants needed hospital treatment. If he went to a co-operating hospital, he paid nothing, and the cost was borne by the Association. If he went to a non-co-operating (or Poor-law) hospital, he was reimbursed by the Association for any charges that he had to pay. Mr. Lesser added that if only people realised the advantages of the scheme, applications for membership would be unlimited.

### A FIVE FIGURE INCOME IN FIVE YEARS.

THE fifth year of the East Lancashire Workpeople's Hospital Fund has proved more successful than ever, and a total of over £13,000 has been raised, or three times as much as was subscribed during the first year of the fund's short existence. We note with pleasure that the committee records a substantial increase in the number of subscribers among the professional and business classes, and the growing readiness of tradesmen to supplement the contributions of their staffs. Shops, banks and offices, no less than workshops and factories, participate in the scheme, which is a great encouragement to other centres like Leeds, or even London, where a similar effort is at an earlier stage of growth. There is no doubt that, if and when times of prosperity return, the recent principle of mass contributions will transform voluntary hospital finance. Those who then will look back to pre-war days will wonder how the hospitals maintained themselves, and will note that London was the last to seek to apply the principle to its very amorphous population. The leaven is working here also, however slowly, and the progress made in London is perhaps the most interesting fact to watch in hospital finance at present.

### DR. ROGER BIRDWOOD.

THE death of Dr. Roger Alan Birdwood, late medical superintendent of Infectious Hospitals, London, removes a distinguished medical reformer who did much to secure the public safety in regard to infectious disease. A Cambridge and Guy's man, after qualifying he entered the service of the Metropolitan Asylums Board, and then took part in the Zulu campaign, for which he received the medal and clasp. On his return to Gravesend Hospital, where he became house surgeon, he advocated and secured a separate ward for children, and in 1884 became medical superintendent of hospital ships under the Metropolitan Asylums Board. There he inaugurated the removal of all small-pox patients from their homes to the ships, in spite of some opposition, and succeeded in reducing the mortality and cost of treatment. In 1892 he successfully tackled the emergency of planning, furnishing and starting the North-Eastern Fever Hospital at Tottenham in the course of a few weeks, and after presiding over it for five years did the same service for the Park Fever Hospital at Hither Green, where he remained till 1915. He advised that these hospitals should be used to provide medical instruction, gave evidence before the Commission on Vaccination, and recommended the recognition of conscientious objectors and the payment of the vaccination fee to the medical man attending the mother of the child at birth. His evidence helped to gain the acquittal of Mr. Chisholme on a charge of manslaughter, and Dr. Birdwood always regarded the accused as a victim of religious and professional intolerance. This independence and his readiness to leave the laurels to others diminished the recognition that he had earned by his own work. But when he retired the Board added five years to his period of service in acknowledgment of his achievements.

### THE LEEDS CONTRIBUTION SCHEME.

MR. T. F. BRAIME is pegging away at his scheme to organise regular contributions to the Leeds Infirmary from the business houses of the city. A representative company of business men has listened to an address from him in the Lord Mayor's parlour. He described an amended scheme by which the collieries are to pay into a pool one farthing a ton, and the workmen a penny a week; each section would thereby raise a sum of £15,000. On the other hand, Mr. Braime reported an amazing state of affairs among the business firms, 831 of which subscribed among them only £750. With these some progress has been made, and 180 firms have joined the contribution scheme. Mr. Braime explained, in answer to a question, that the cost of running the Leeds hospitals and convalescent funds is £175,000 a year under the voluntary system. Under the State it would be £300,000, or, in other words, the product of a rate of two shillings in the pound. This argument was probably impressive to an audience which took the existence of hospitals for granted and supposed that it was somebody else's business to support them. A beginning has been made, however, and the history of every similar scheme shows that this is the chief difficulty. If Mr. Braime will not lose heart and will continue his efforts, the example of the contributing firms will spread and the scheme become an accepted habit.

### THE BUGBEAR OF MAINTENANCE.

EVEN the most enthusiastic supporter of weekly contributions to hospitals would have hesitated to assert that this source of income would not suffer from unemployment. Yet this can be the case. A notable example is at hand from Edinburgh, where the Royal Infirmary's income from the League of Subscribers has increased by more than £2,000 during the past year. It is now for the first time over £20,000. The interesting fact is that, while the subscriptions from men in certain firms has indeed diminished, the local losses are more than made up by the growth of the scheme. It has now been taken up by the staffs of schools and in Government and other offices. To-day there are 72,000 members in Greater Edinburgh and 22,000 in country districts, and this means that there have been added 9,000 new members during the year. These are eloquent figures. They suggest, above all, that if the money can be raised to extend the infirmary, which has a waiting list larger than it has ever been, there need be no ultimate anxiety about maintenance. Such a promise should encourage wealthy benefactors as nothing else can do.

### POOR LAW INFIRMARIES AT BIRMINGHAM.

THE Chief Medical Officer of the Birmingham Union in his annual report draws attention to the number of cases admitted from the voluntary hospitals. Compared with the figures of 1921, the total transfers have increased from 522 to 1,084. He expects the transfers to diminish for the interesting reason that general practitioners are now communicating direct with the Guardians' hospitals for the admission of urgent cases. These hospitals have also come to the relief of patients in neighbouring Unions who require special treatment and have been unable to find admission to the voluntary hospitals. In other words, the Poor Law Hospitals, the best of which are admittedly well-equipped and up-to-date, are playing an increasingly useful part in the health service of the country, and deal with much besides the chronic ailments of old age. The readiness of general practitioners and their patients to seek admission to them is also interesting, but it remains an anomaly that even paying patients continue to suffer legal disabilities for taking advantage of the provision that everyone is anxious for them to use.

### MARYLEBONE LEAGUE OF WORKERS.

THE Earl of Athlone took farewell of the Middlesex Hospital, of which he has been chairman prior to his departure for South Africa, at a luncheon given to him by the Mayor of St. Marylebone on behalf of the associated hospitals of the borough. The Marylebone Hospitals League of Workers attained a membership of six thousand within three months of its foundation, and though the progress of such schemes is slow in London, time will probably instal the League in most of the business houses in the borough, especially as membership has given more than an obligation to subscribe in the demonstrations and lectures that have been eagerly attended by the members. The staff of the Middlesex Hospital combined to present three pieces of plate to the departing chairman, who has held office during some of the most difficult years. It was a happy idea of the Mayor's to give this luncheon, at which the representatives of many business houses in Marylebone were present. The whole scheme, as we recorded at the beginning, was an interesting and bold experiment—a real step in co-ordination which made the hospitals educational centres also for the supporters of the League. The Mayor declared that the support received from business interests in the borough was far too small, but, luckily, it is against all precedent for such schemes not to develop.

### NEW HOSPITAL AT HIGH WYCOMBE

THE recent opening of the High Wycombe War Memorial Hospital on Marlow Hill, by Field-Marshal Sir William Robertson, has been the result of a great effort. The whole cost of £35,000 has been met, and the hospital has started free of debt with thirty-six beds in place of the small number at the disposal of the cottage hospital. A sign of the times is the endeavour, from the very beginning of the new institution, to secure regular weekly contributions from the staffs of the local manufacturers. The staple industry of the town is furniture and chair-making, and much of the equipment has been generously contributed. If this spirit continues the difficulty of maintenance will be solved, for the organisation of weekly contributions is the modern form of partial endowment, and without this a new institution may be totally handicapped. A hospital that is also a War Memorial especially deserves this form of maintenance, for the spirit that built it should survive to carry on the trust from year to year by a common effort in the neighbourhood.

### DOMESTIC STAFFS AND THE DOLE.

THE Hospital Committee of the Chertsey Council, after discussing the difficulty of obtaining a domestic staff at the hospital, sent a letter to the local Members of Parliament and to the Ministry of Labour stating that in their opinion unemployment benefit should not be given to women and girls when domestic work was obtainable if they would accept it. There can be no doubt that the dole is much abused, and that work for which its recipients are qualified is often refused while the benefit is obtainable. There is undeniably a prejudice against domestic work to-day, when the ideal is an office stool or a cashier's desk or a position behind the counter. But many of the alleged—and largely imaginary—disadvantages of domestic work do not exist in institutions where hours, modern appliances, conveniences, bathrooms, and communal life are all provided. When, therefore, institutions cannot get domestic staffs, a prejudice must exist, and it is time to consider how far the dole is giving it artificial support.

### ROCKEFELLER HELP FOR WALES.

THE Welsh Medical School at Cardiff is to receive a gift of £14,000 from the Rockefeller Foundation, New York. This very important gift, no less valuable for the recognition that it confers than for the financial help, is to be used to build laboratories and to improve the clinical facilities of the medical unit of the Royal Infirmary. It is understood that the Foundation was influenced when making the gift to some extent by the unit system of teaching adopted in the School during the final years of study. Heroic efforts have been made to place the school and the infirmary upon their feet, and this magnificent donation is the reward of the endeavour made in Cardiff and throughout Wales for many years past.

